# Ocular and Mucocutaneous Sequelae among Survivors of Stevens-Johnson Syndrome and Toxic Epidermal Necrolysis in Togo

**DOI:** 10.1155/2019/4917024

**Published:** 2019-01-30

**Authors:** Bayaki Saka, Abla Séfako Akakpo, Julienne Noude Teclessou, Garba Mahamadou, Abas Mouhari-Toure, Kossi Dzidzinyo, Adam Nouhou Diori, Nidain Maneh, Sabin Prince-Agbodjan, Koussake Kombaté, Komi Balo, Kissem Tchangai-Walla, Palokinam Pitché

**Affiliations:** ^1^Dermatology Unit, Sylvanus Olympio Teaching Hospital, University of Lomé, Togo; ^2^Dermatology Unit, Campus Teaching Hospital, University of Lomé, Togo; ^3^Dermatology Unit, Kara Teaching Hospital, University of Kara Ophthalmology, Togo; ^4^Ophthalmology Unit, Campus Teaching Hospital, University of Lomé, Togo

## Abstract

**Aim:**

The aim of this study was to assess ocular and mucocutaneous sequelae among SJS/TEN survivors and identify risk factors of ocular sequelae.

**Patients and Method:**

Late complications among SJS/TEN survivors were assessed using 2 methods: a retrospective assessment of medical records only or a retrospective assessment of medical records and physical examination of survivors who were contacted by phone.

**Results:**

Between January 1995 and December 2017, 177 cases of SJS/TEN (138 cases of SJS, 29 cases of TEN, and 10 cases SJS/TEN overlap) were admitted into two university hospitals of Lomé (Togo). There were 113 women and 64 men, with an average age of 31.7±13.0 years (range: 5 to 80 years). The most used drugs were antibacterial sulfonamides (35.6%) and nevirapine (24.3%). HIV serology was positive in 68 (59.1%) of the 115 patients tested. Sixty-four (52,5%) of the 122 patients, who had been examined by an ophthalmologist during the acute stage, had acute ocular involvement, which was mild in 27.9% of patients, moderate in 13.1%, and severe in 11.5%. We recorded 17 deaths (i.e., three cases of SJS, 12 of TEN, and two of SJS/TEN overlap), including 11 cases of HIV infected patients. Of the 160 SJS/TEN survivors, only 71 patients were assessed 6 months after hospital discharge. Among them, forty-three (60.6%) patients had sequelae. Concerning mucocutaneous sequelae, the main lesions were diffuse dyschromic macules (38.0% of patients) and ocular sequelae were dominated by decreased visual acuity (14.1% of patients). In multivariate analysis, exposure to sulfadoxine (odds adjusted ratio = 5.95; 95%CI= [1.36-31.35]) and moderate (adjusted odds ratio = 5.85; 95%CI = [1.23-31.81]) or severe (adjusted odds ratio = 48.30; 95%CI = [6.25-1063.66]) ocular involvement at acute stage were associated with ocular sequelae.

**Conclusion:**

Ocular and mucocutaneous sequelae are common in SJS/TEN survivors. Exposure to sulfadoxine and severity of acute ocular involvement are risk factors of ocular sequelae.

## 1. Introduction

Stevens-Johnson syndrome (SJS) and Toxic Epidermal Necrolysis (TEN) are serious drug induced dermatologic occurrences which can be life-threatening at the acute stage [1]. They are considered the most severe types of cutaneous adverse reactions to drugs, with high morbidity and mortality rates [1, 2]. Chronic physical complications among SJS/TEN survivors have been reported in the skin, eyes, oral and genital mucous membranes, gastrointestinal tract, teeth, kidneys, and lungs [3–7]. Ocular sequelae are dreadful and can lead to blindness; meanwhile, the mucocutaneous sequelae can alter the functional and social prognosis [3]. A previous research identified exposure to sulfadoxine as a factor associated with the severity of the ocular involvement at the acute stage SJS/TEN [8]. The aim of this study was to assess the ocular and mucocutaneous sequelae at the chronic phase and to identify the risk factors for ocular sequelae in sub-Saharan Africa.

## 2. Patients and Method

Our study included all SJS/TEN patients treated into two university hospitals (Sylvanus Olympio and Campus) of Lomé (Togo) between January 1995 and December 2017. To assess the long-term ocular and mucocutaneous complications among SJS/TEN survivors, we used two methods: (i) a retrospective assessment of medical records only or (ii) a retrospective assessment of medical records and physical examination of survivors (who had never returned for control 6 months after hospital discharge), that we contacted by phone between September and November 2017. In this study, we defined sequels as ocular and mucocutaneous complications or symptoms which appears or persisted 6 months after the initial episode of toxidermia.

### 2.1. Retrospective Medical Records

A retrospective assessment of medical records was performed to gather information concerning the acute stage of SJS/TEN and the medical follow-up by a dermatologist or ophthalmologist. We used a detailed questionnaire designed for this study to assess medical status including the following information: demographic data (age; sex), medical history (medication use, type of toxidermia, data regarding hospitalization due to SJS/TEN, acute ocular involvement, the time between ocular involvement and local ocular care, number of local ocular cares/day), paraclinical data (HIV serology), outcomes (death), and medical follow-up data (ocular and mucocutaneous sequelae among SJS/TEN survivors examined by a dermatologist or ophthalmologist, 6 months after hospital discharge). With this method, we collected long-term complications of 50 patients. At the acute stage, the severity of the toxidermia and the ocular involvement were assessed using the Bastuji-Garin et al. [9] and the Power and al. [10] classifications, respectively. According to the Power classification, the ocular involvement is said to be

- mild when there is an eyelid edema and/or conjunctival hyperemia and/or chemosis alone;
- moderate when there is a membranous conjunctivitis and/or superficial punctate keratitis and/or infiltration or ulcer of the cornea;
- severe when there is a conjunctival hemorrhage and/or symblepharon and/or unhealed superficial punctate keratitis and/or decreased visual acuity.

### 2.2. Retrospective Medical Records and Physical Examination

Except retrospective assessment of records to gather information in the acute stage of SJS/TEN, a physical examination was done by a well-experienced dermatologist and ophthalmologist through cutaneous, mucous membrane, and ophthalmic examination to assess long-term complications. With this method, we included 21 SJS/TEN survivors contacted by phone.

### 2.3. Statistical Analysis

Data entry was done using Epidata version 3.1 software. These data were then exported to the R software (version 3.3.4) with which the analysis was carried out. For descriptive analysis, quantitative variables were described by means (and extremes), whereas qualitative variables were described by frequencies (absolute and relative). The comparison of these means and frequencies enabled to establish existing links between the study covariables and the presence of ocular sequelae. The Pearson's Chi-square test or the Fisher's test were used to compare qualitative variables, whereas, Student test was used to compare quantitative variables. Finally, all the covariables associated with the existence of ocular sequelae with p-value ≤ 0.25 were included in a logistic regression model to identify associated factors. The significance threshold was set at 5%.

## 3. Results

During the study period, a total of 177 SJS/TEN cases (138 for SJS, 29 for TEN and 10 overlapping SJS/TEN) were reviewed. There were 113 women and 64 men, with an average age of 31.7±13.0 years (range: 5 to 80 years). The main used drugs were antibacterial sulfonamides (35.6%) and nevirapine (24.3%) (Table 1). HIV serology was positive in 68 (59.1%) of the 115 patients tested. Sixty-four (52.2%) of the 122 patients who had an ophthalmological examination at the acute stage had an ocular involvement which was mild in 27.9% of cases, moderate in 13.1% of cases, and severe in 11.5% of cases. Of the 177 SJS/TEN patients, 17 deaths were recorded in the acute stage (i.e., three cases of SJS, 12 of TEN, and two of overlapping SJS/TEN) including 11 HIV infected patients. Of the 160 SJS/TEN survivors, only 71 patients were assessed 6 months after hospital discharge. Among them, forty-three (60.6%) patients had sequelae. The average time of follow-up was 6.2 ± 3.1 years (range: 6 months to 13 years). Cutaneous sequelae were predominantly diffused dyschromic macula and the ocular sequelae were mostly decreased visual acuity and symblepharon (Table 2); some patients might have presented several types of sequelae. The dyschromic sequelae were either hyper or hypochromic macula or poikiloderma. The two cases of vaginal adhesions were observed in two girls aged 12 and 14 years. In multivariate analysis, exposure to sulfadoxine (odds adjusted ratio=5.95; CI 95%=[1.36 – 31.35]) and moderate (odds adjusted ratio=5.85; CI 95%=[1.23 – 31.81]) or severe (odds adjusted ratio=48.30; CI95%=[6.25 – 1063.66]) ocular involvement in the acute stage increased the risk of ocular sequelae (Table 3). Furthermore, 5 patients without ocular involvement at the acute stage of the toxidermia presented with ocular sequelae.

## 4. Discussion

We aimed to characterize the long-term ocular and mucocutaneous complications among SJS/TEN survivors and identify risk factors of ocular sequelae. It confirmed the frequency of ocular and mucocutaneous sequelae among patients who survived SJS and TEN. However, we identified that exposure to sulfadoxine and the severity of the ocular involvement at the acute stage are risk factors for ocular sequelae. In this study, we defined sequels as ocular and mucocutaneous complications or symptoms which appears or persisted 6 months after the initial episode of toxidermia. The long average time of follow-up, 6.2 ± 3.1 years (range: 6 months and 13 years), constitutes the strength of this study and can be explained by the fact that some patients were called and summoned several years after leaving the hospital.

Our study clearly revealed that 38% of SJS/TEN survivors suffer from long-term cutaneous complications such as diffuse dyschromic macules (38.0% of the patients), pruritis (20% of the patients), hypertrophic scars (2.8% of the patients), nail dystrophy (1.4% of the patients), and hair loss (1.4% of the patients). These frequencies are significantly lower than those reported by other authors which ranged from 72 to 77% [4, 5, 11]. The long average time of follow-up and the skin pigmentation of our patients (black skin) might explain the under-diagnosis of hyperchromic sequelae. This skin pigmentation might also explain the absence of photosensitivity cases and the fact that the sequelae were dyschromic in our series, contrary to the hyperchromic and hypochromic sequelae which were respectively reported in the studies of Fellahi and al. [4] and Sheridan and al. [11]. The young age of the patients and the female predominance made cutaneous sequelae the major source of discomfort for aesthetic reasons. The cause of hair loss could probably be telogen effluvium, however further research is needed to study hair pathology in an important number of SSJ/TEN survivors. Moreover, the hyperchromic scars reported in our series are related to the peculiarity of black skin which has the tendency to develop cheloids. At last, other long-term cutaneous complications like eruptive naevi, anetodermia, cutaneous calcifications, or increased sweating reported in other series [3, 4, 12, 13] were absent in our study.

Our study also revealed that 36.6% of survivors of SJS/TEN had ocular sequelae, dominated by reduced visual acuity (14.1% of the patients), followed by symblepharon (12.7% of the patients), photophobia (12.7% of the patients), and dry eyes (9.9% of the patients). Dry eyes syndrome which is the main ocular long-term complication reported in literature [4, 5, 12–14] was found in 9.9% of our patients, probably not often researched. Ocular sequelae can go as far as blindness since 2.8% of our survivors of SJS/TEN had become blind. This frequency represents one-third of the 8% reported by Gueudry and al. [14]. In Fellahi and al. series [4], no case of blindness was reported. Our recommendation that all patients must be followed by an ophthalmologist arises from the frequent ocular complications revealed in our study and is also in accordance with the recent review on the treatment of acute and chronic ophthalmic involvement in SJS/TEN [15], indicating that there is an important window of opportunity to prevent major ocular sequelae following index hospital discharge even with minor or unnoticed ophthalmic abnormalities.

In this study, we identified that exposure to sulfadoxine and the severity of the ocular involvement at the acute stage are risk factors for ocular sequelae. The severity of the ocular involvement at the acute stage is already known as a factor conditioning the frequency and severity of ocular sequelae after skin scarring [14, 16, 17]. Exposure to sulfadoxine being a factor associated with the risk of ocular sequelae is not surprising since it is associated with the severity of the ocular involvement at the acute stage in sub-Saharan Africa [8]. Jongkhajornpong et al. [18] found that exposure to antibiotics and to nonpharmaceutical triggers was a risk factor for ocular sequelae. Furthermore, our study did not find an association between ocular sequelae and HIV infection, unlike these series [18] where HIV infection protects against ocular sequelae. Finally, five patients without ocular lesions or symptoms in the acute stage of the SJS/TEN had ocular sequelae. This is in accordance with previous reports in the series of Gueudry and al. [14] and Fellahi and al. [4], where 5 and 3 cases, respectively, without ocular involvement in the acute stage presented with long-term ocular complications.

## 5. Limitations

The main limitations of this study were the retrospective character and the small size of the study population.

## 6. Conclusion

This study confirmed that ocular and mucocutaneous sequels are common in SJS/TEN survivors. However, exposure to sulfadoxine and severity of acute ocular involvement are risk factors for ocular sequels. Hence, these results suggest a shift in the approach to SJS/TEN survivors by recognizing their challenging physical complications and recommending that all survivors must be followed by a dermatologist and ophthalmologist, whether symptoms are absent or minimal at discharge from the hospital.

## Figures and Tables

**Table 1:** Drugs implicated in the cause of SJS/TEN.

**Drugs used**	**No. of cases**	**(**%**)**
Antibacterial sulfonamides	63	35.6
*Sulfadoxine*	*37*	*20.9*
*Sulfamethoxazole*	*26*	*14.7*
Nevirapine	43	24.3
Antiepileptics	14	7.9
Amino-penicillin	7	3.9
Analgesics	6	3.4
Nonsteroidal anti-inflammatory drugs	6	3.4
Antibacterial sulfonamides /Nevirapine	4	2.3
Traditional drugs*∗*	3	1.7
Chinese drug of undetermined nature	2	1,1
Not determined	29	16.4
**Total**	**177**	**100**

*∗*: mixed decoctions of medicinal plants and ingredients.

**Table 2:** Ocular and mucocutaneous sequels in 71 SJS/TEN survivors.

	Number	%
**Cutaneous sequelae**	**30**	**42.3**
*Diffuse dischromic macules*	27	38.0
*Pruritis *	6	20.0
*Hypertrophic scars*	2	2.8
*Nails dystrophy *	1	1.4
*Hair loss (telogen effluvium) *	1	1.4

**Mucous sequelae**	**6**	**8.5**
*Labial adhesions*	4	5.6
*Vaginal adhesions*	2	2.8

**Ocular sequelae**	**26**	**36.6**
*Decreased visual acuity*	10	14.1
*Symblepharon*	9	12.7
*Photophobia with watering eyes*	9	12.7
Dry eyes	8	9.9
*Ocular adhesions*	3	4.2
*No light perception (blindness)*	2	2.8
*Superficial punctate keratitis*	1	1.4
*Entropion*	1	1.4

**Table 3:** Risk factors of ocular sequelae among SJS/TEN survivors.

	Sequelae	Univariate analysis				Multivariate analysis		
	Yes, n=26	No, n=45	OR	95% CI	P value	aOR	95% CI	P value
**Age (years)**								
< 35 years, n (%)	21 (80.8)	27 (60.0)	2.80	[0.94-9.60]	0.0775			
**Sex**								
Female, n (%)	19 (73.1)	27 (60.0)	1.81	[0.65-5.43]	0.2692			

**Type of toxidermia**					0.6929			
SJS, n (%)	20 (76.9)	38 (84.4)	1	-				
Overlap SJS/TEN, n (%)	2 (7.7)	3 (6.7)	1.27	[0.16-8.25]				
TEN, n (%)	4 (15.4)	4 (8.9)	1.90	[0.41-8.83]				
**HIV serology, n=59** **∗**								
Negative, n (%)	12 (46.2)	21 (46.7)	0.78	[0.27-2.24]	0.6423			

**Ocular involvement at acute stage**					**0.0057**			**0.0005**
None, n (%)	5 (19.2)	25 (55.6)	1	-		1		
Mild, n (%)	7 (26.9)	13 (28.9)	2.69	[0.72-10.76]		3.55	[0.86-16.66]	0.0874
Moderate, n (%)	6 (23.1)	6 (13.3)	5.0	[1.15-23.54]		5.85	**[1.23-31.81]**	**0.0303**
Severe, n (%)	8 (30.7)	1 (2.2)	40.0	[5.66-835.63]		48.30	**[6.25-1063.66]**	**0.0013**
**Used drugs, n=68** **∗** **∗**								
Sulfadoxine, n (%)	8 (30.7)	12 (26.7)	4.22	[1.17-17,59]	**0.0331**	5.95	**[1.36-31.35]**	**0.0230**
Sulfamethoxazole, n (%)	4 (15.4)	9 (20.0)	1.34	[0.30-5,61]	0.6814			
Nevirapine, n (%)	9 (34.6)	20 (44.4)	1.49	[0.51-4,33]	0.4599			
NSAIDs + analgesics, n (%)	3 (11.5)	3 (6.7)	2.86^e^10^7^	[1.10^e^10^−71^-..]	0.9901			
**Time between ocular involvement and local ocular care, n=40** **∗** **∗** **∗**								
>7 days, n (%)	5 (19.2)	3 (6.7)	1.89	[0.39-10,50]	0.4333			
**Number of sessions of local ocular care/day, n=33** **∗** **∗** **∗** **∗**								
>1 day, n (%)	13 (50.0)	17 (37.8)	0.38	[0.02-4,42]	0.4522			

^1^Student test; SJS: Stevens-Johnson syndrome; TEN: toxic epidermal necrolysis; OR: odds ratio; aOR: adjusted odds ratio; *∗*: HIV was known in 59 patients; **∗**: drug used found in 68 patients; *∗ ***∗***∗*: delay specified in 40 patients; *∗∗ ***∗***∗*: number of sessions specified 33 patients; NSAIDs: nonsteroidal anti-inflammatory drugs.

## Data Availability

Extracted data are with the authors and available for sharing upon request.

## Abbreviations

SJS: Stevens-Johnson syndrome
TEN: Toxic epidermal necrolysis.

## References

[1] Saka B., Barro-Traoré F., Atadokpédé F. A. (2013). Stevens-Johnson syndrome and toxic epidermal necrolysis in sub-Saharan: a multicentric study in four countries. *International Journal of Dermatology*.

[2] Dodiuk-Gad R. P., Chung W.-H., Valeyrie-Allanore L., Shear N. H. (2015). Stevens–Johnson syndrome and toxic epidermal necrolysis: An update. *American Journal of Clinical Dermatology*.

[3] Lee H. Y., Walsh S. A., Creamer D. (2017). Long-term complications of Stevens-Johnson syndrome/toxic epidermal necrolysis (SJS/TEN): the spectrum of chronic problems in patients who survive an episode of SJS/TEN necessitates multidisciplinary follow-up. *British Journal of Dermatology*.

[4] Fellahi A., Zouhair K., Amraoui A., Benchikhi H. (2011). Stevens-Johnson and Lyell syndromes: mucocutaneous and ocular sequels in 43 cases. *Annales de Dermatologie et de Venereologie*.

[5] Olteanu C., Shear N. H., Chew H. F. (2018). Severe physical complications among survivors of Stevens-Johnson and toxic epidermal necrolysis. *Drug Safety*.

[6] Yang C.-W., Cho Y.-T., Chen K.-L., Chen Y.-C., Song H.-L., Chu C.-Y. (2016). Long-term sequelae of Stevens–Johnson syndrome/toxic epidermal necrolysis. *Acta Dermato-Venereologica*.

[7] Saeed H., Mantagos I. S., Chodosh J. (2016). Complications of Stevens-Johnson syndrome beyond the eye and skin. *Burns*.

[8] Saka B., Dzidzinyo K., Akakpo S. (2018). Factors associed with the severity of acute ocular involvement in Stevens-Johnson syndrome and toxic epidermal necrolysis in Sub-saharan Africa. *Annales de Dermatologie et de Venereologie*.

[9] Bastuji-Garin S., Rzany B., Stern R. S., Shear N. H., Naldi L., Roujeau J.-C. (1993). Clinical classification of cases of toxic epidermal necrolysis, Stevens-Johnson syndrome and erythema multiforme. *Archives of Dermatology*.

[10] Power W. J., Ghoraishi M., Merayo-Lloves J., Neves R. A., Foster C. S. (1995). Analysis of the acute ophtalmic manifestations of the erythema multiforme/Stevens-Johnson syndrome/toxic epidermal necrolysis disease spectrum. *Ophthalmology*.

[11] Sheridan R. L., Schulz J. T., Ryan C. M. (2002). Long-term consequences of toxic epidermal necrolysis in children. *Pediatrics*.

[12] Oplatek A., Brown K., Sen S., Halerz M., Supple K., Gamelli R. L. (2006). Long term follow-up of patients treated for toxic epidermal necrolysis. *Journal of Burn Care & Research*.

[13] Gelfer A., Rivers J. K. (2007). Long term follow-up of a patient with eruptive melanocytic nevi after Stevens-Johnson syndrome. *Archives of Dermatology*.

[14] Gueudry J., Roujeau J.-C., Binaghi M., Soubrane G., Muraine M. (2009). Risk factors for the development of ocular complications of Stevens-Johnson syndrome and toxic epidermal necrolysis. *Archives of Dermatology*.

[15] Kohanim S., Palioura S., Saeed H. N. (2016). Acute and chronic ophthalmic involvement in Stevens–Johnson syndrome/toxic epidermal necrolysis: a comprehensive review and guide to therapy. II: Ophthalmic disease. *The Ocular Surface*.

[16] Kim D. H., Yoon K. C., Seo K. Y. (2015). The role of systemic immunomodulatory treatment and prognostic factors on chronic ocular complications in Stevens-Johnson and toxic epidermal necrolysis. *American Journal of Ophtalmology*.

[17] López-García J. S., Rivas Jara L., García-Lozano C. I., Conesa E., De Juan I. E., Murube del Castillo J. (2011). Ocular features and histopathologic changes during follow-up of toxic epidermal necrolysis. *Ophthalmology*.

[18] Jongkhajornpong P., Lekhanont K., Siriyotha S., Kanokrungsee S., Chuckpaiwong V. (2017). Factors contributing to long-term severe visual impairment in stevens-johnson syndrome and toxic epidermal necrolysis. *Journal of Ophthalmology*.

